# Rationales and arguments behind the adoption of self-selection of nonprescription medicines in Denmark

**DOI:** 10.1186/s40545-020-00226-2

**Published:** 2020-07-08

**Authors:** Solveig Nordahl Jacobsen, Simone Eggert Møller-Jensen, Sofia Kälvemark Sporrong

**Affiliations:** Social and Clinical Pharmacy, Department of Pharmacy, Faculty of Health and Medical Sciences, University of Copenhagen, Universitetsparken 2, 2100 Copenhagen, Denmark

**Keywords:** Nonprescription medicine, Pharmaceutical policy, Pharmacy regulation, Argumentation analysis

## Abstract

**Background:**

Pharmacies in Europe have undergone considerable changes in their regulation over the last decades, also regarding nonprescription medicines (NPMs). In 2001, selected NPMs were released for sale outside pharmacies in Denmark. To ensure consumer safety, it was decided that NPMs must be stored behind the counter. In 2018, an amending act came into force, which allowed self-selection of NPMs. The purpose of this study was to examine the rationales and related arguments, including their validity and relevance, behind the policy on self-selection of NPMs in Denmark.

**Methods:**

A qualitative study design, combining document analysis and individual interviews with key stakeholders, was used. Legislative documents were retrieved from the Parliaments’ homepage. Interviewees were recruited through purposeful sampling. Interviews were analyzed using directed content analysis. Rationales and supporting arguments were identified, thematized and analyzed as to their validity and relevance.

**Results:**

In total, 24 stakeholders (including political parties) were represented in the documents, whereof 7 were interviewed. Ten supported the new policy and 13 were against; 1 was on both sides. Six rationales and 9 supportive arguments were found. The advocates’ main rationale was increased accessibility and arguments related to freedom of choice and discretion. The opponents’ main rationale for not adopting the policy was consumer safety and arguments related to perception of NPMs and counseling. The validity and relevance were questionable in both advocates’ and opponents’ arguments, yet slightly better in the case of the opponents’. Although not mentioned in the documents, economic interests were probably behind some stakeholders’ position.

**Conclusion:**

The formal rationale behind the adoption of self-selection of NPMs was increased accessibility. However, bearing in mind the rationales and their supporting arguments, economic interests and previous changes within the sector, it could be argued that an underlying rationale behind adopting the policy was to liberalize the Danish pharmacy sector even further.

## Background

Pharmacies in Europe have undergone considerable changes in their regulation over the last two decades. Today, many European pharmacy sectors have been liberalized, among them the Norwegian and Swedish sectors [1, 2]. A study from 2015, found that as opposed to a decade before, in 16 out of 19 European countries, pharmacy ownership was no longer restricted to pharmacists; Denmark was one of only three countries, where it still was [2].

Besides ownership, the European pharmacy sectors vary, among other things, in their regulation of nonprescription medicines (NPMs). In the United Kingdom, some NPMs (General Sales List medicines) have been approved for non-pharmacy sales since the 1960’s [3]. In Sweden, non-pharmacy sales including self-selection, i.e. that customers can grab the product from the shelf for themselves, of NPMs have been allowed since 2009 [4, 5]. In Norway, non-pharmacy sales of NPMs were introduced in 2003, and self-selection from 2011 [6, 7].

In Denmark, the area of NPMs has been regulated numerous times throughout the last two decades [8]. As a consequence of political pressure from the right-wing to liberalize the pharmacy sector, and in general eliminate monopolies, selected NPMs were released for non-pharmacy sale in 2001 [9, 10]. However, all NPMs had to be stored inaccessible to customers, such as behind a counter, independent of sales place. On 1 January 2018, an amending act, proposed by the right-wing government, came into force, making self-selection of NPMs legal. With this adoption, selected NPMs became available for self-selection, allowing them to be sold from shop areas and not only at the counter, both in and outside pharmacies. The amendment did not alter the selection of NPMs in pharmacy or non-pharmacy sale respectively. The rationale given by the Ministry of Health was that giving consumers the possibility to assess which NPM to purchase supported accessibility [8, 11].

### The setting

The Danish pharmacy sector is highly regulated. Consumers should have no more than 15 km to a pharmacy, which is ensured by an equalization system providing subsidies to pharmacies in sparsely populated areas [12]. Moreover, Danish prices on generic medicines are among the lowest in Europe due to biweekly competitive tendering applying to all pharmacies. In 2018, NPMs made up 11%, approximately 1.4 billion DKK, of the total Danish drug expenditure. Pharmacy only NPMs accounted for 3 % and NPMs approved for non-pharmacy sale 8 % (5 % sold at pharmacies, 3 % sold outside pharmacies) [13]. Only prescription medicines are reimbursed.

**Fig. 1:**
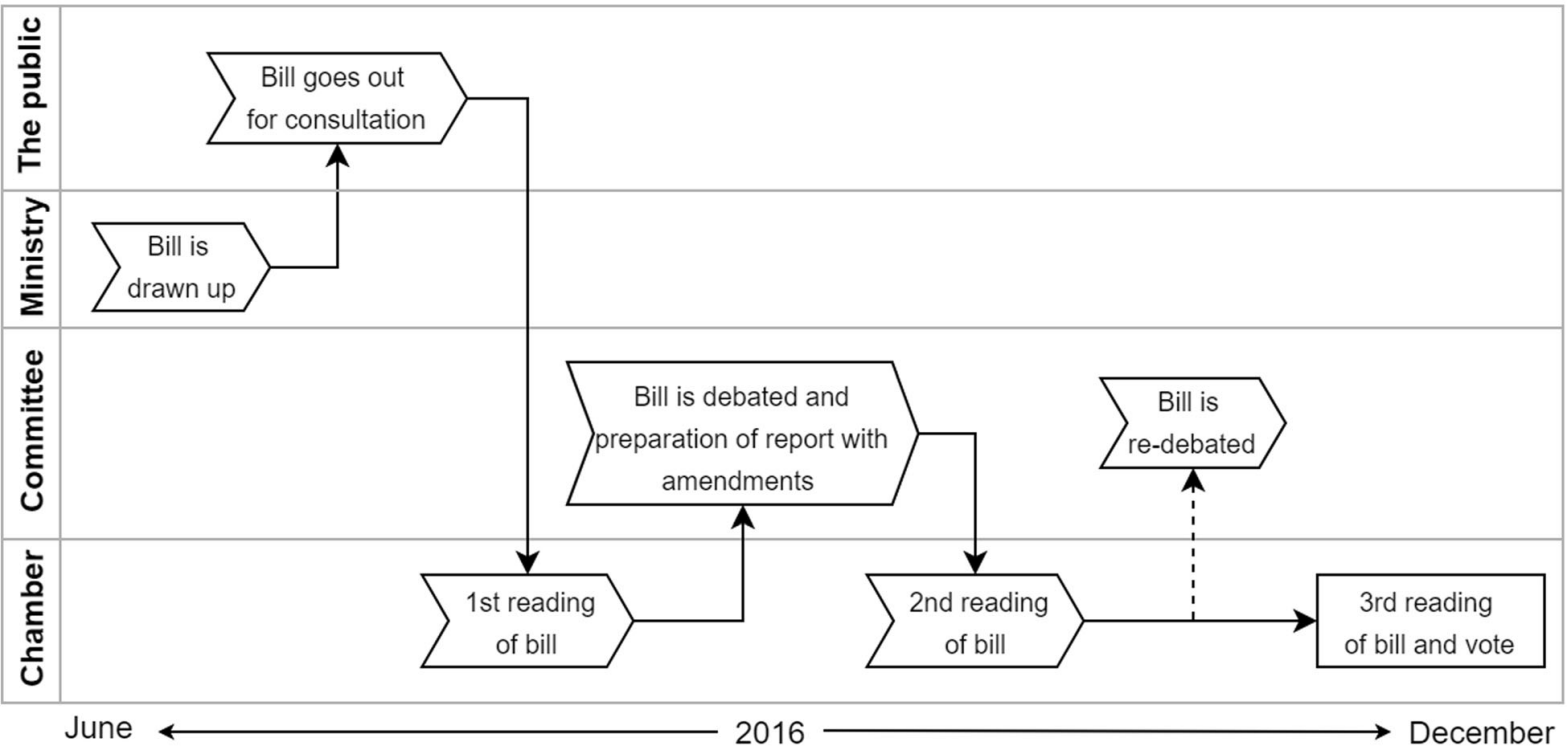
Illustration of the legislative process of the bill in question in the Danish Parliament. The figure should be read from left to right. The figure is inspired by the illustration on the Danish Parliament’s homepage [14]

The different steps of the legislative process in Denmark are illustrated in Fig. 1. Bills are proposed by the Government and passed by the Parliament after being read three times in the Chamber. Bills are drafted by the concerned ministry and sent out for public consultation before being read in the Parliament. In this phase, stakeholders have the opportunity to comment on the bill.

In the case of self-selection of NPMs, the complete bill included two separate proposals; one suggesting the adoption of self-selection of NPMs and another suggesting amendment of an existing pharmacy watch service, determining opening hours of the pharmacies. Although separate, the two propositions were put forward in one bill in October 2016 entitled *“Better accessibility to medicines by establishment of the pharmacies’ watch service and self-selection of nonprescription medicines”* [8]. All stakeholders involved acknowledged the need for the amended watch service, therein supporting the adoption of this part of the bill. The second part about self-selection of NPMs was, however, much debated. Experts within the field did not see the need for the scheme, as only placement of NPMs was amended, raising question to what was behind the proposal.

### Aim

The aim of this study was to examine the rationales and related arguments, including their validity and relevance, behind the policy on self-selection of NPMs in Denmark.

## Methods

Two qualitative data collection methods were combined: Document analysis and individual interviews. The document analysis served the purpose of examining the rationales and arguments presented during the processing of the bill. Individual semi-structured interviews with key stakeholders were carried out to elaborate and complement these findings. In addition, the validity and relevance of the arguments were assessed through argumentation analysis.

### Qualitative document analysis

Documents covering the legislative process of the bill concerning self-selection of NPMs were identified on the Parliament’s homepage [15]. Following Bowen, the document analysis was carried out by the first and second author, in three steps: (1) skimming, whereby the researchers roughly sorted out documents not relevant to the research question, (2) thorough reading, in which the researchers examined the documents thoroughly and marked facts, rationales and arguments, but also unsubstantiated statements, (3) analysis, in which the researchers found patterns within the data [16].

All documents identified were included in the document analysis. Material from expert meetings and formality documents on timetables and agendas were however excluded due to missing documentation (expert meetings) and after skimming the content (timetables/agendas). Documents included for thorough reading and analysis (*n* = 28) are listed in Additional file 1. They reflected statements of all political parties represented in the Danish Parliament, one public authority, and 14 interest organizations. The first and second author carried out the thorough reading separately. For each document a worksheet was filled out in which passages related to the research question were organized. All passages were assigned with a tentative theme based on the content.

In order to provide an overview of a) the rationales and b) the arguments for the rationales, a thematization process took place. In several consensus meetings, the tentative themes were discussed, and rationales, i.e. stakeholders’ reasoning behind their position on the scheme, as well as arguments connected to the rationales, were identified. The latter were assigned to the rationales they supported/opposed. It was noted which rationales and arguments that were in support of or in opposition to the proposal.

### Individual semi-structured interviews

The individual interviews were semi-structured, meaning that they had a number of themes to be covered, but the order and ranking of the questions depended on the interviewee’s answers [17]. Except for the interview guide for the Ministry of Health, which was focused on the process (not presented here), all interview guides comprised five overall topics: 1) accessibility to medicines, 2) the scheme after implementation, 3) the legislative process, 4) rationales and arguments and 5) interests. All topics in the interview guide originated from the document analysis but were tailored for each interviewee depending on the specific stakeholder’s response in the public consultation phase. Questions were mostly open-ended. The structure of the interviews enabled new questions to emerge continuously and the possibility for asking clarifying questions. The interview guides were adjusted in an inductive manner, using the concept of learning from interview to interview [17].

The recruitment strategy was purposeful sampling [18]. Stakeholders were identified from the document analysis. Stakeholders were included if their attitude towards the topic was either unexpected or strong, or if another stakeholder mentioned them as important. In addition, an attempt was made to obtain an equal number of opponents and advocates. Individuals representing the selected organizations were contacted by email. The emailed individual was provided with the opportunity to pass the email on to another more appropriate person within the organization. Based on the above criteria, nine stakeholders were contacted. Of these, seven agreed to participate: The Ministry of Health and six interest organizations.

The interviews were carried out in April and May 2018 by the first and second author. The interviews were audio-recorded and transcribed verbatim, and lasted on average 43 min (range 21 min - 56 min). All interviews were held at the work place of the interviewee. The participants were offered to view their own interview transcript. No changes were made.

Interview data was analyzed using directed (deductive) content analysis. Predefined themes emanated from the document analysis [19]. Relevant passages that did not fit into a predefined theme, were organized in new themes (inductively). The analysis was initially conducted by the first and second author separately, then discussed and finalized in consensus meetings with all authors.

Quotations from the interviews are referred to by the abbreviations of the stakeholders listed in Table 1.

**Table 1:** Overview of stakeholders, whether they were interviewed and their position on the bill

Stakeholders	Interviewed	Supporting	Opposing
**Political parties**			
Liberal Alliance (LA)^a^	No	x	
The Alternative (ALT)	No		x
The Conservative Party (KF)^a^	No	x	
The Danish People’s Party (DF)^a^	No	x	
The Danish Social Democrats (S)^b^	No^c^	x	
The Liberal Party (V)^a^	No	x	
The Red-Green Alliance (EL)	No		x
The Socialist People’s Party (SF)	No		x
The Social Liberal Party (RV)	No		x
**Public authorities**			
The Ministry of Health (MH)	Yes	x	
**Organizations**			
Danish Generic and Biosimilars Medicines Industry Association (IGL), ‘the generic industry’	Yes		x
Danish Regions (DR)	No		x
Local Government (LG)	No	x	
Pharmadanmark (PD)^d^	No	x	x
The Association of Danish Pharmacies (ADP), ‘the pharmacy owners’	Yes		x
The DaneAge (DAA)	No		x
The Danish Association of Pharmaconomists (DAP)	Yes		x
The Danish Association of the Pharmaceutical Industry (Lif), ‘the originator industry’	Yes	x	
The Danish Chamber of Commerce (DCoC)	Yes	x	
The Danish Consumer Council (DCC), ‘the consumer organization’	Yes		x
The Danish Medical Association (DMeA)	No		x
The Danish Nurses Organization (DNO)	No		x
The Danish Patient Association (DPA)	No^c^	x	
The Danish Patient Safety Association (DPSA)	No		x

^a^Government parties (KF, LA, V) and government supporting party (DF)

^b^Argued against adoption of self-selection of NPMs but voted for adoption of the bill

^c^Declined interview

^d^Argued for self-selection of NPMs at pharmacies but not at retail outlets

### Argumentation analysis

To assess the validity and relevance of the arguments put forward, an argumentation analysis was carried out by the first and second author subsequent to the document and interview analyses. As described by Björnsson et al., for an argument to have high probative force it requires both validity and relevance [20]. The validity and relevance of the arguments were assessed according to the rationales they supported. An argument was assessed as valid if its conclusion appeared true (e.g. in relation to research) and trustworthy. An argument had relevance if it was relevant for the rationale it was supposed to support [20].

## Results

In total, 9 political parties, one public authority and 14 interest organizations argued for or against the new policy (see Table 1).

The Government parties (and hence the Ministry of Health), their supporting party, and the biggest opposition party, the Danish Social Democrats, supported the policy. The latter argued against the proposition even though they at the end voted for adoption. Opponents of the policy were the other opposition parties. In terms of interest organizations, most were against, with the originator industry, the Danish Chamber of Commerce, the Local Government (that did not have any comments) and the Danish Patient Association being the exceptions. The labor union for pharmacists, Pharmadanmark, argued both for and against.

The advocates and opponents each presented rationales with supportive arguments supporting or opposing, respectively, adoption of self-selection of NPMs. In the analysis process 6 rationales and 9 supportive arguments were identified (see Table 2).

**Table 2:** An overview of rationales and supportive arguments

	Supportive arguments
**Primary rationales**	
Increased accessibility to NPMs	Increased freedom of choice
	Increased discretion
Decreased consumer safety	Shift in perception of NPMs
	Decreased counselling
**Assistant rationales**	
Increased/reduced costs for consumers	Display, advertising and impulse buying
	Increased price competition
No need for scheme	No demand from consumers
Works in neighboring countries	No complications in Sweden or Norway
	Populations similar to that in Denmark
**Rationales not presented in documents**	
Economic interests	Increased sales of medicines
	Shift from generic to brand NPMs

First, the primary rationales from the two sides are presented together with their supportive arguments, including their validity and relevance. Then, assistant rationales including supportive arguments are presented. Lastly, a rationale, not presented in the documents, about stakeholders’ economic interests, is described.

### Primary rationale – increased accessibility to NPMs

As presented by the Ministry, the rationale behind the complete bill was increased accessibility to medicines for the public, which was also the rest of the advocating stakeholders’ primary rationale for adopting self-selection of NPMs. In the documents, advocates stated that the scheme would increase the public’s accessibility to NPMs, which in turn would facilitate the process of buying NPMs. The Social Liberal Party challenged the rationale, saying that there would be no harm in NPMs being a little difficult to access.

Accessibility to NPMs was defined in different ways. Opponents and advocates agreed that good accessibility to NPMs was defined as easy physical access to the products. However, the operationalization of the concept varied: factors mentioned included geographical distance, opening hours, access for physically disabled people, connection between pharmacies and emergency doctors, and waiting times. In addition to this, opponents mentioned access to counselling, while advocates highlighted the importance of discrete access and having options to choose from.

#### Freedom of choice

Advocates from the political sphere emphasized that the scheme would result in increased freedom of choice for the consumers, thus supporting the rationale on accessibility. The Danish Patient Association supported what they called a consumer-centered way of thinking; while the political opposition countered that there was no point in providing something the consumers did not demand. The stakeholders argued whether or not the consumers had the ability to manage their own illnesses and thus use their freedom to choose between NPMs. The originator industry drew on experience from earlier liberalizations in Denmark and Norway, stating that consumers had managed to remain rational in their purchase of NPMs. The consumer organization emphasized the importance of consumers’ possibility to manage their medication, but found, similar to the association of Pharmaconomists, self-selection of NPMs a risk to consumers, as they would not have the sufficient knowledge.

*“It is naive to think that consumers can choose medicines by themselves. It requires knowledge. You are a novice when you enter a new area, and you need advice and guidance.” – DCC*

In the interviews, some opponents claimed that the argument concerning freedom of choice was invalid as:

*“In our opinion, it is a bit of a pseudo argument that the consumer is presented to a greater freedom of choice. It will not be the full range [of products] anyway.” – DAP*

Analyzing the argument, self-selection of NPMs may, in theory, increase the customers’ freedom to choose between NPMs. With the scheme, customers are able to grab the preferred NPM by themselves. The possibility to choose is, however, not a result of the scheme, as this was possible prior to the scheme, just through staff. Advocates argued for more options to choose from, but as mentioned by opponents of the scheme, freedom of choice depends on a broad display of products. As this is not an evident outcome of the scheme, the arguments’ validity becomes questionable. In terms of relevance, the argument only seems relevant if increased freedom to choose falls within the definition of accessibility.

#### Discretion

Discretion was the other of the advocates’ arguments supporting the rationale on accessibility. In the documents they argued that self-selection of NPMs would result in increased discretion, making the purchase situation more comfortable for the consumers as they would be spared making an oral inquiry during a purchase. Opponents acknowledged the lack of discretion as a problem. However, they argued that the scheme would not be the solution to this problem, as public accessibility would require clear signage.

In the interviews, the originator industry elaborated their consultation response, stating that full discretion was impossible to obtain, but self-selection of NPMs was a step in the right direction. The consumer organization agreed that pharmacies could improve discretion, but found the argument irrelevant on NPMs, as one should not feel ashamed when purchasing these medicines.

*“No, because I think it is nonsense in relation to these products. There is nothing to be ashamed of buying nicotine gum.” – DCC*

The validity of discretion as an argument can be questioned, as the scheme simply postpones the oral inquiry until later in the sales situation, at least at pharmacies where staff is obligated to provide counselling. In addition, the customer still has to pay at the counter where the product will be displayed, questioning the relevance of this argument as well [21, 22].

### Primary rationale - decreased consumer safety

Opponents presented decreased consumer safety as the primary rationale for not adopting self-selection of NPMs, as they claimed that the scheme would challenge this. There were different perspectives on how consumer safety could be worsened if the scheme came through. Opponents were concerned that self-selection of NPMs would result in inappropriate use, e.g. increased consumption and misuse. For example, they referred to Sweden, where the Swedish Medical Product Agency withdrew paracetamol tablets from non-pharmacy sales in 2015 due to an increase in poisoning inquires [23]. However, the originator industry advocated that no earlier experience pointed towards overuse or misuse of NPMs.

#### Shift in perception of NPMs

In the documents, nearly all opponents of the scheme worried that self-selection of NPMs would cause a shift in the consumers’ perception of NPMs, which could lead to inappropriate use of NPMs, thereby supporting their primary rationale.

The importance of medicines being viewed as special products rather than harmless everyday consumer goods was emphasized. The originator industry was the only advocating organization that commented on this, stating that the risk of NPMs turning into everyday consumer goods was small. This was elaborated in the interview, where it was stated that other consumer products could be potentially dangerous as well, but still could be purchased without thorough guidance.

*“Other product groups [mentioning cars as an example] are potentially dangerous, but you can still get them without counselling. Counselling on medicines is not provided every time anyway.” – Lif*

When asked, the consumer organization, replied that singlehanded pressure from industry pushed the perception of NPMs towards everyday consumer goods. The pharmacy owners expressed a concern about consumers using medicines that they did not require.

*“Something happens when things become accessible. /… / There are people who will start using medicines that they would not have asked their doctor for.” – ADP*

A study on NPMs following the liberalization in 2001 showed that the Danish population had a poor understanding of NPMs and medicines in general, and in what way they differed from, e.g. dietary supplements [24]. By introducing self-selection of NPMs, there is a possibility that the difference between NPMs and everyday consumer goods is wiped out even more, making the opponents’ argument valid. The concern about the possible shift in perception of NPMs was also addressed in 2001. Back then, storage behind the counter was established to avoid this risk. It is debatable whether a shift in perception occurred with the liberalization in 2001, or if it is probable to happen now, strengthening the relevance of this supporting argument.

#### Counselling

Multiple opponents, including all those interviewed, emphasized the importance of counselling by pharmacy staff as support for their rationale on consumers safety. The pharmacy owners and the Danish Association of Pharmaconomists stressed that self-selection of NPMs at pharmacies would complicate the staff’s ability to carry out sufficient counselling, and that the dialogue may switch from a symptom-to-product approach to a product-to-symptom approach. Some advocates acknowledged the problem of insufficient counselling. When asked about counselling and consumers’ awareness of their need for this, the originator industry was skeptical towards the counselling provided by pharmacy staff in general, saying that the problem of insufficient counselling would be present anyway. The Danish Chamber of Commerce said that inadequate counselling had nothing to do with self-selection of NPMs but with having NPMs outside pharmacies, referring to the change in 2001.

*“You already have that problem [inadequate counselling] at the pharmacy. Counselling is not thorough enough to uncover all these things [compliance] at the pharmacy either.” – Lif*

There are also studies supporting the argument on counselling. For example, in a study of Swedish pharmacies from 2001, 60% more medicine-related problems were detected and solved when NPMs were sold over the counter, compared to when sold from shop areas with self-selection [25]. Studies from Germany and Denmark also supported the importance of counselling, concluding that respectively 18 and 21% of customers requested the wrong NPM or used them inappropriately [26, 27]. In a study from the United States, the counselling from a pharmacist led to prevention of potential side effects of NPMs, linked to NPMs customers had the intention of buying, in 7% of encounters [28]. Considering that customers, with self-selection of NPMs, grab the products themselves, the risk of purchasing an inappropriate product increases.

Deduced from this, the argument on counselling is somewhat valid in relation to self-selection of NPMs at pharmacies in Denmark, supporting the opponents’ rationale for not adopting the scheme due to consumer safety. Contrary, as advocates argued, the possibility of counselling is still present at pharmacies for those in need of it. However, it can be questioned, if those in need are aware of their need; this is supported by the aforementioned studies. At retail outlets, counselling was absent prior to the scheme as well, making the arguments about counselling less valid in relation to these sales places. Hence, the problem of insufficient counselling arose back in 2001 when selected NPMs were released for free trade, and the relevance of counselling is therefore low in relation to the scheme in question.

### Assistant rationale – costs for consumers

Another rationale identified was related to costs and how the scheme could influence consumers’ economy. Both sides used this rationale but differed on whether they thought the scheme would lead to increased or reduced costs for consumers.

#### Display, advertising and impulse buying

Opponents were concerned that self-selection would make NPMs subject to impulse buying and thus lead to an increased expenditure for consumers. Advocates countered these claims, drawing on experiences from earlier liberalizations in the area of NPMs in Denmark, Norway and Sweden, saying that no irrational or increased expenditure had been observed. The pharmacy owners criticized this conclusion, as the experience was based on sales of NPMs in general and not exclusively on NPMs available for self-selection. Additionally, they pointed out that sales of NPMs had increased by 42% from 2008 to 2015 in Sweden.

Some opponents, including the generic industry and the pharmacy owners, stated that self-selection of NPMs would lessen price competition due to the power of advertising influencing the range of NPMs exposed and hence sale of generics. They were concerned that in-store marketing might favor expensive brand NPMs and affect the consumers’ choice of product, making purchases more expensive and less rational.

*“If there is nothing to choose from then it is hard to talk about comparability and things like that.”* – ADP

It has been shown that large increases in shelf space can increase brand sales [29]. Further, Sclar et al. concluded that customers saved money due to pharmacist counselling, as the customers often demanded products seen in commercials and pharmacy staff recommended a generic, cheaper one [28]. The validity of the argument concerning display, advertising and impulse buying is thus less valid in relation to pharmacies due to counselling. However, retail outlets usually do not stock more than one substitutable product, i.e. the branded product, thus increasing the validity of the argument in this setting. Further, experience from Norway indicates that the introduction of self-selection shifts the sales of NPMs from pharmacies towards retail outlets, resulting in more consumers purchasing medicines in places without counselling, increasing the validity of the argument even more [30]. As no rules have been laid down regarding range of NPMs displayed or in-store marketing in correlation to the scheme, the argument is of high relevance.

#### Price competition

Advocates stressed that self-selection of NPMs would intensify the price competition, as the prices would be exposed to the consumers, thereby resulting in lower prices and reduced costs for consumers. In the interview, the consumer organization stated that price competition was already strong, resulting in low prices on NPMs.

*“I do not believe in price reductions due to self-selection of nonprescription medicines. It is simply utterly naive if you believe that self-selection will lower the prices. There is already so much competition on those medicines, and they are ridiculously cheap.”* – DCC

When asked to comment on the practical execution of price competition in the interviews, advocates referred to normal market economics. However, this argument was not that important.

*“The economy was something we mentioned, but it was by no means something that mattered.”* – Lif

In theory, exposure of the products to the consumers may increase price competition. Again, since no rules have been established on the range of products to be displayed, the price competition may be limited. The originator industry acknowledged that this argument was a theoretical one. Despite the argument being valid, the relevance is low, as no measures are taken to ensure the preconditions for price competition. In addition, as opponents mentioned, advertising may influence the purchase of NPMs rather than the price, as these products are already rather cheap, at least according to the consumer organization.

### Assistant rationale – no need for scheme

A rationale stressed by opponents was the absence of need for self-selection of NPMs. Hence, they questioned if consumers even needed the scheme, and if not, what would be the benefit. They argued that the lack of demand from consumers supported their rationale. The generic industry stressed that the originator industry and the Danish Chamber of Commerce were the only organizations asking for the scheme. In the interview the originator industry agreed that there had not been a marked demand for self-selection of NPMs, but neither a demand for the contrary.

*“It is natural that there is not a demand for something like self-selection of nonprescription medicines. As a citizen without knowledge in the field, you simply do not pay attention to it.”* – Lif

Hence, both opponents and advocates acknowledged that no demand for self-selection of NPMs was put forward by consumers, making the argument valid. However, it is not a matter of course that amendments have to be in demand or needed to be carried out, questioning the relevance of this argument in this debate. Still, a non-existent need or demand affects to what extent a new policy is reasonable to adopt, making this point somewhat relevant.

### Assistant rationale - works in neighboring countries

Through the interviews, experiences from the neighboring countries Sweden and Norway were often referred to. Almost all advocates spontaneously mentioned this without being asked. The rationale, as presented by advocates, seemed to be that having schemes in Sweden and Norway that work without significant complications is solely enough to adopt it in Denmark. They emphasized the similarities between the Danish population and the populations of the two other countries, arguing that the scheme hence could be implemented in Denmark without difficulty.

It can be questioned how transferable these experiences are to the Danish system, and thus the validity of this argument. The pharmacy sectors in Sweden and Norway differ from that in Denmark in regard to ownership and organization [31, 32]. In Norway, wholesale dealers own most of the pharmacies, e.g. making the risk of favoring specific brands greater compared to Denmark. Also, the proposed scheme of self-selection of NPMs differs from that in Sweden and Norway in what NPMs are included and how to execute the scheme [4–7]. Moreover, the geography differs – both Norway and Sweden have large areas that are sparsely populated, hence the nearest pharmacy can be far away. This is not true for Denmark. However, looking at the populations of Denmark, Sweden and Norway, it can be argued that they are similar in sense of their perception of medicines and ability to manage medication, thereby making the comparison somewhat relevant.

### Rationale not presented in documents – economic interests

Economic interests, i.e. economic wins or losses as a consequence of implementing the scheme, were almost not mentioned in any of the documents studied. However, when asked, the generic industry, the originator industry, the Danish Chamber of Commerce and the Danish Association of Pharmaconomists all acknowledged economic interests as a rationale in relation to self-selection of NPMs, which influenced their attitude towards the scheme. The originator industry stated that they had an interest in additional sales of medicines, however, with avoidance of medicine abuse. The generic industry stated that the scheme exclusively considered the financial well-being of the originator industry, and expressed a concern that it would result in them loosing earnings to originator companies, as the generic industry depended on the pharmacies to inform consumers about generic alternatives. This is a valid argument as the brand NPMs are well-known by the customers and more displayed both in and outside of pharmacies, e.g. through commercials. The consumer organization pointed at lobbyism performed by the originator industry in the processing of the bill.

Regarding economic interests of the pharmacy sector, the pharmacy owners denied having any economic interests, referring to the economic system for the pharmacy sector. This statement is questionable, as sales of NPMs released for free trade is one of the ways to increase the earnings of the individual pharmacy [12, 33]. Furthermore, according to the other interviewed organizations, pharmacy owners did have an economic interest. The Danish Association of Pharmaconomists, whose members are mostly employed at pharmacies, recognized the economic importance for the pharmacies. Based on these factors, it is expected that economic interests also affected pharmacy owners’ attitude towards self-selection of NPMs.

## Discussion

The advocates’ primary rationale behind adopting self-selection of NPMs was increased accessibility, while opponents’ primary rationale against adoption was a decrease in consumers’ safety. Moreover, the opponents reasoned that there was no need or demand for the scheme and the advocates stated that experience from neighboring countries was proof enough to adopt the proposition. Also, there were different views on whether the scheme would lead to increased costs for consumers or money savings.

All organizations interviewed, except for the consumer organization, could be said to have some economic interest in relation to self-selection of NPMs. This has probably affected their attitude towards the scheme, and should be taken into consideration when examining the rationales and arguments presented. Most of the rationales and arguments presented sought to consider the consumers exclusively. However, it appears that other factors were at stake as well, e.g. economic earnings for the originator industry and the pharmacy owners when presenting arguments on price competition and counselling respectively.

This study shows that analysis of arguments can nuance pharmaceutical policy analyses. Arguments are the fundamental units of the political decision-making process. Democratic decisions, like the approval of policies, may ideally be viewed as an effect of the better argument. However, even in cases with extensive evidence the influence of evidence depends on the way it is used in arguments addressed to decision-makers and stakeholders [34]. From the analysis it can be concluded that, the validity and relevance were questionable in both advocates’ and opponents’ arguments in relation to their respective rationales, yet from an overall assessment slightly better in the case of the opponents’. Still the scheme came through, showing that the decision-making process is not one where the better argument wins.

### Framing the proposal

There was no demand from the public to introduce self-selection of NPMs. Instead, the Government defined a problem with the existing accessibility, stating that it was inadequate in terms of consumers’ possibility to assess which NPMs to purchase. They proposed a solution to the problem, this being establishment of self-selection of NPMs. Hence self-selection of NPMs became a solution to a problem that did not exist in the public. This strategy is referred to as ‘framing’ and is used, among other things, to describe policymakers’ way of defining a problem to fit a specific solution, thus creating an acceptable reason for performing a reform, even though there is no direct demand or need identified. In other words, an issue is “framed” in terms that suit a specific solution [35]. Further, as Hiilamo and Kangas argue, policymakers must frame their proposals in a way that appears to be the best solution to a problem, and they have to be wrapped in ‘*normatively acceptable ways*’, referring to widely accepted values, such as freedom, social justice and equality [36]. Taking the wording of the bill into consideration, the word ‘accessibility’ is used in the title and the Government placed great emphasis on ‘freedom to choose’ in their arguments. Both terms have positive connotations, playing on the three aforementioned values; values that are core in right-wing policy, but also difficult for opponents to counter. If the rationale, stating that the scheme worsens consumer safety, is true, then there is a choice between increased accessibility and consumer safety. You would, however, not see a government framing a proposal as ‘worsened consumer safety’. The Government thus framed the proposal in the most ‘acceptable’ way.

### Parallels to the liberalization in 2001

From an overall policy viewpoint, these results should be seen in the light of Danish pharmacy policy historically. In their article ‘Advocacy coalitions and pharmacy policy in Denmark – Solid cores with fuzzy edges’, Larsen et al. examined the debate on a potential liberalization of the pharmacy sector in the years 1996–2001 [10]. In their analysis they used the Advocacy Coalition Framework (ACF) and particularly the hypotheses of advocacy coalitions, policy change, and learning across coalitions. Even though almost two decades have past (and ACF calls for at least a decade of perspective [37]), and that the policy change in the current study was minor in comparison, there are still similarities between the results of the two studies.

The two coalitions identified 1996–2001 had core beliefs either focused on public control or on market mechanisms. The same two coalitions can, roughly, be seen in the current study. However, in the study by Larsen et al., the core beliefs of the current debate – increased accessibility on the market side and patient safety on the public control side – were back then both rationales *for* the public control coalition. Accessibility as discussed then was e.g. access to NPMs, but not least access to medicines in rural areas. Some of the presumed negative consequences of a reform, as argued by the public control coalition were the same as now, e.g. irrational use of NPMs, based on increased access and marketing as well as lack of professional counselling.

The market control coalition, back then, argued for less regulation in order to open up for more competition. This was supposed to lead to, amongst other, a more consumer-oriented market, which can be compared with the arguments on freedom of choice, discretion and price competition in the current debate. It seems that the market coalition belief today is more focused on freedom of choice than competition. Competition was only mentioned in the current debate in relation to prices; i.e. competition as such was not seen as a mechanism for better accessibility (apart from prices). Hence, it seems as the whole debate has turned somewhat away from the former market mechanism core belief towards the public control core belief.

Larsen et al. explains the prerequisite for the suggested changes (‘external shock’ in ACF terms [37]) to be the ‘general spread of market-oriented ideas’ at the time [10]. Market-oriented ideas have also earlier been seen to influence pharmacy policy, but that has also been criticized [1, 38, 39]. As mentioned above, this kind of market-oriented core belief was not as explicit in the arguments in the current study. However, using experiences from Sweden and Norway as an argument is implicitly pointing at this, as the reforms in these countries were clearly inspired by market-oriented ideas [31, 39, 40].

The initiatives, both regarding the discussion in 1996–2001 and that today, came from the more market oriented coalition (freedom of choice rather than equality and consumer safety). The discussions back then did not lead to a liberalization of the Danish pharmacy sector, apart from NPMs being sold outside pharmacies. However, the current, minor, reform could be seen as a step in the direction of a market-oriented sector, i.e. a liberalization, but ‘hidden’ behind the argument of neighboring (liberalized) countries. Also in this case the change was not as big as wished for by advocates. Similarly to 2001, the discussions in this case did not lead to a full ‘implementation’ of the proposal. The policy ended up being something in between both coalitions, as executive orders, presented after the bill was approved, introduced several comprehensive restrictions in regard to the scheme. These were patient safety related restrictions such as supervision and further placement requirements [22]. The Social Democrats, an advocate of the public control coalition, ended up voting for a limited market-oriented reform. Here it should be considered that the proposal suggesting adoption of self-selection of NPMs was put forward together with a proposal suggesting amendment of the pharmacies’ watch service, altering pharmacy opening hours, which all stakeholders acknowledged the need for. It is possible that pooling the proposals enabled the adoption of the scheme. The Danish Social Democrats might have found the proposition on the watch service more important to adopt than rejecting the one on self-selection of NPMs, which they argued against.

### Limitations

The documents were accessed through the Parliament’s homepage, thus making the retrievability of the study high. However, there were documents, which were not accessible and therefore neither have been read nor incorporated in the analysis. Then again, none of these documents were mentioned by interviewees.

The Danish Social Democrats and the Danish Patient Association did not agree to participate in interviews, hence information might be missing on their arguments and especially why the party changed position during the processing of the bill. No political parties were interviewed, however, one can argue that the ministry takes the perspective of the governmental parties.

It has to be taken into consideration that the interviewees were representatives of the organizations; hence, there is a possibility that other people within the organization had other views or more knowledge on the area. To avoid this, the email was directed to the individual who signed the consultation response. Additionally, the organizations chose the interviewee.

Some arguments that were used by only one or few stakeholders were not included in this study, e.g. packaging of NPMs.

Argumentation analysis implies an interpretation of rationales and arguments, which is a limitation for the method as such. Specifically the discrepancies in the operationalization of accessibility, made it difficult to assess the validity and relevance of some arguments. The assessment of validity and relevance in the argumentation analysis is also affected by the inherent limitations of those concepts, namely the limited available empirical knowledge to assess the validity of a claim and the normative assumptions about what is relevant.

## Conclusion

The main rationale behind adopting self-selection of NPMs in Denmark was better accessibility to medicines. This was also the advocates’ primary rationale, while the opponents’ primary rationale for not adopting the scheme was decreased consumer safety. Nothing indicated that the adoption of the amendment came about because of the advocates’ arguments being superior to the opponents’, discarding a decision-making process where the better argument wins. The use of argumentation analysis enabled a nuanced analysis of the pharmaceutical policy, where validity and relevance of both parties’ arguments were questionable, yet better for opponents. This also has to be seen in the light of economic interests.

It is possible that by using framing, the Government was able to propose a solution to a problem, for which there was no obvious need or demand. By applying the theory of advocacy coalition framework, parallels can be drawn between the scheme in question and the liberalization in 2001, with two coalitions advocating different belief systems; public control or market mechanisms.

Bearing in mind the recent changes within the pharmacy sector in Denmark, the imitation of the Swedish and Norwegian systems, and the findings from this study, adopting self-selection of NPMs might be another step towards a more liberalized pharmacy sector in Denmark. As market orientation gains ground on public control in pharmaceutical policy, i.e. business versus health care perspective, the pharmacy sector is moved further away from the health care sector.

Analyzing arguments within pharmaceutical policy, including which stakeholders use the arguments, makes it possible to further understand mechanisms behind these policies and the ideologies behind.

## Supplementary information

**Additional file 1.** Documents used in document analysis. Documents used in document analysis in a chronological order. Each document has been provided with a recognition code (code), the title in original language, the author (using abbreviations), publication date, and the date that the document was debated in the Parliament.

## Data Availability

The interview transcripts generated and analyzed during the current study are not publicly available due to that anonymity cannot be fully ensured. However, the intermediate analysis and documents are available from the corresponding author on reasonable request.
