# Supplementary material for: Rationales and arguments behind the adoption of self-selection of nonprescription medicines in Denmark
Source: J Pharm Policy Pract. 2020 Jul 8;13:29. doi: 10.1186/s40545-020-00226-2 (PMC7341604; doi:10.1186/s40545-020-00226-2)
Supplement: Supplementary file 1 — Additional file 1. Documents used in document analysis. Documents used in document analysis in a chronological order. Each document has been provided with a recognition code (code), the title in original language, the author (using abbreviations), publication date, and the date that the document was debated in the Parliament. [file 40545_2020_226_MOESM1_ESM.docx]

# Additional file 1: Documents used in document analysis

Documents used in document analysis in a chronological order. Each document has been provided with a recognition code (code), the title in original language, the author (using abbreviations), publication date, and the date that the document was debated in the Parliament.

| Code | Title of document | Document type | Author | Publication date | Date debated |
| --- | --- | --- | --- | --- | --- |
| A.01 | Håndkøbslægemidler i selvvalg – Muligheder for selvvalg af visse håndkøbslægemidler på apotek og i detailvirksomheder | Report | Danish Medicines Agency | Jun 2016 | 06 Oct 2016 |
| B.01 | Høringssvar: Apotekerforeningen | Consultation response | ADP | 18 Jul-02 Sep 2016 | 21 Sep 2016 |
| B.02 | Høringssvar: Lægeforening | Consultation response | DMeA | 18 Jul-02 Sep 2016 | 21 Sep 2016 |
| B.03 | Høringssvar: Lif | Consultation response | Lif | 18 Jul-02 Sep 2016 | 21 Sep 2016 |
| B.04 | Høringssvar: Dansk Erhverv | Consultation response | DCoC | 18 Jul-02 Sep 2016 | 21 Sep 2016 |
| B.05 | Høringssvar: Patientforeningen | Consultation response | DPA | 18 Jul-02 Sep 2016 | 21 Sep 2016 |
| B.06 | Høringssvar: IGL | Consultation response | IGL | 18 Jul-02 Sep 2016 | 21 Sep 2016 |
| B.07 | Høringssvar: Dansk Selskab for Patientsikkerhed | Consultation response | DPSA | 18 Jul-02 Sep 2016 | 21 Sep 2016 |
| B.08 | Høringssvar: Ældre Sagen | Consultation response | DAA | 18 Jul-02 Sep 2016 | 21 Sep 2016 |
| B.09 | Høringssvar: Farmakonomforeningen | Consultation response | DAP | 18 Jul-02 Sep 2016 | 21 Sep 2016 |
| B.10 | Høringssvar: Dansk Sygeplejeråd | Consultation response | DNO | 18 Jul-02 Sep 2016 | 21 Sep 2016 |
| B.11 | Høringssvar: Forbrugerrådet Tænk | Consultation response | DCC | 18 Jul-02 Sep 2016 | 21 Sep 2016 |
| B.12 | Høringssvar: Danske Regioner | Consultation response | DR | 18 Jul-02 Sep 2016 | 21 Sep 2016 |
| B.13 | Høringssvar: pharmadanmark | Consultation response | PD | 18 Jul-02 Sep 2016 | 21 Sep 2016 |
| B.14 | Høringssvar: Kommunernes Landsforening | Consultation response | LG | 18 Jul-02 Sep 2016 | 21 Sep 2016 |
| C.01 | Høringsnotat | Consultation report | MH | 21 Sep 2016 | 21 Sep 2016 |
| D.01 | Skriftlig fremsættelse (2016): 2016/1 SF.L L 38 | Introduction of bill | MH | 06 Oct 2016 | 06 Oct 2016 |
| E.01 | Forslag til Lov om ændring af lov om apotekervirksomhed og lov om lægemidler: 2016/1 LSF 38 [1] | Bill | MH | 06 Oct 2016 | 06 Oct 2016 |
| F.01 | L 38 Forslag til Lov om ændring af lov om apotekervirksomhed og lov om lægemidler: 1. behandling | 1st reading | The Danish Parliament | 27 Oct 2016 | 27 Oct 2016 |
| Code | **Title of document** | **Document type** | **Author** | **Publication date** | **Date debated** |
| G.01 | Risici ved selvvalg af lægemidler (lovforslag 38) | Written inquiry | ADP | 11 Nov 2016 | - |
| H.01 | L 38 Forslag til Lov om ændring af lov om apotekervirksomhed og lov om lægemidler: Spørgsmål | Q&A | Health Committee & MH | 10 Oct-2 Dec 2016 | 06 Dec 2016 |
| I.01 | Bilag: SUU L 38 – svar på spm. 29 - Ændringsforslag til Forslag til Lov om ændring af lov om apotekervirksomhed og lov om lægemidler | Amendment 1 | ALT | 21 Nov 2016 | 13 Dec 2016 |
| I.02 | Bilag: SUU L 38 – svar på spm. 39 - Ændringsforslag til Forslag til Lov om ændring af lov om apotekervirksomhed og lov om lægemidler | Amendment 2 | EL | 02 Dec 2016 | 13 Dec 2016 |
| J.01 | Betænkning over Forslag til lov om ændring af lov om apotekervirksomhed og lov om lægemidler | Report | Health Committee | 06 Dec 2016 | 06 Dec 2016 |
| K.01 | L 38 Forslag til Lov om ændring af lov om apotekervirksomhed og lov om lægemidler: 2. behandling | 2nd reading | The Danish Parliament | 13 Dec 2016 | 13 Dec 2016 |
| L.01 | Ændringsforslag til 3. behandling af Forslag til Lov om ændring af lov om apotekervirksomhed og lov om lægemidler | Amendment 3 | ALT | 15 Dec 2016 | 16 Dec 2016 |
| M.01 | L 38 Forslag til Lov om ændring af lov om apotekervirksomhed og lov om lægemidler: 3. behandling af lovforslag nr. L 38 | 3rd reading | The Danish Parliament | 16 Dec 2016 | 16 Dec 2016 |
| N.01 | Forslag til Lov om ændring af lov om apotekervirksomhed og lov af lægemidler: 2016/1 LSV 38 | Adopted bill | MH | 16 Dec 2016 | - |
| O.01 | Lov om ændring af lov om apotekervirksomhed og lov om lægemidler: LOV nr 1736 af 27/12/2016 | Amending act | H.M. Queen Margrethe II of Denmark | 27 Dec 2016 | - |
